# Outsourcing cleaning services increases MRSA incidence: Evidence from 126 english acute trusts

**DOI:** 10.1016/j.socscimed.2016.12.015

**Published:** 2017-02

**Authors:** Veronica Toffolutti, Aaron Reeves, Martin McKee, David Stuckler

**Affiliations:** a Department of Sociology, University of Oxford, Oxford, UK; b International Inequalities Institute, London School of Economics and Political Science, Houghton Street, London, UK; c Department of Public Health and Policy, London School of Hygiene and Tropical Medicine, London, UK

**Keywords:** Outsourcing, Hospital acquired infections, Hospital cleaning, Contracting-out

## Abstract

There has been extensive outsourcing of hospital cleaning services in the NHS in England, in part because of the potential to reduce costs. Yet some argue that this leads to lower hygiene standards and more infections, such as MRSA and, perhaps because of this, the Scottish, Welsh, and Northern Irish health services have rejected outsourcing. This study evaluates whether contracting out cleaning services in English acute hospital Trusts (legal authorities that run one or more hospitals) is associated with risks of hospital-borne MRSA infection and lower economic costs.

By linking data on MRSA incidence per 100,000 hospital bed-days with surveys of cleanliness among patient and staff in 126 English acute hospital Trusts during 2010–2014, we find that outsourcing cleaning services was associated with greater incidence of MRSA, fewer cleaning staff per hospital bed, worse patient perceptions of cleanliness and staff perceptions of availability of handwashing facilities. However, outsourcing was also associated with lower economic costs (without accounting for additional costs associated with treatment of hospital acquired infections).

## Highlights

• Investigation on the association between outsourcing cleaning services and HAI.
• Data on 126 English acute hospital Trust during 2010–2014 were used.
• Outsourcing cleaning services was associated with greater incidence of MRSA.
• Outsourcing was also associated with lower economic costs.

## 1 Introduction

There is a long-standing debate in the United Kingdom about the impact of outsourcing of hospital cleaning services to private sector contractors. Beginning in 1983, cleaning services were one of the first parts of the NHS to be contracted to private providers under HC(8318) “Competitive tendering in the provision of domestic, catering and laundry services”. The then Department of Health and Social Security wanted hospitals to save money and argued that they would “make the maximum possible savings by putting services like laundry, catering and hospital cleaning out to competitive tender. We are tightening up, too, on management costs, and getting much firmer control of staff numbers”(Conservative Party, 1983)._ENREF_1_ENREF_1.

Always controversial, in the 1990s critics linked outsourcing to growing concerns about hospital acquired infections, and in particular, *methicillin-resistant Staphylococcus aureus* (MRSA), which was felt to be especially frequent in the UK (Johnson, 2011, Washer and Joffe, 2006). Media coverage emphasised the role played by “dirty” hospitals (Chan et al., 2010), drawing on evidence of the importance of hospital cleanliness (Dancer, 2009, Dancer, 2008, Davies, 2009, Davies, 2010), patients’ perception of cleanliness (Greaves et al., 2012, Trucano and Kaldenberg, 2007) and frequency of handwashing to preventing infections (Sroka et al., 2010, Stone et al., 2012). There was speculation, and extensive anecdotal evidence, that contractors were seeking to save money, for example by employing fewer staff, with poorer working conditions and hence lower motivation, and were as a result achieving lower levels of cleanliness than the in-house NHS staff they replaced (Davies, 2010). In addition, contracted-out services were considered too inflexible to deal with changing circumstances, including problems with unscheduled cleaning out-of-hours, which might have increased risks of outbreaks (Davies, 2010). Because of these concerns, the Royal College of Nursing called for hospital cleaning to be brought in-house in 2008 (BBC News, 2008) and, later that year, Nicola Sturgeon, then Scottish Health Minister, instructed that this be done in all Scottish hospitals to reduce risks of infection (European Federation of Public Service Unions, 2011)_ENREF_11_ENREF_1, later linking this move with the subsequent fall in cases of *C. difficile* infection (Daily Record, 2011), although this view was not universally accepted, with others linking it to improved antimicrobial stewardship (Nathwani et al., 2012). Outsourcing has also ceased in Wales and Northern Ireland (European Federation of Public Service Unions, 2011). However, these fears were dismissed by others, with the Business Services Association, representing outsourcing companies, arguing that “There is no evidence to suggest that outsourcing cleaning services causes increased rates of infection” (BBC News, 2008)_ENREF_11.

This debate has been handicapped by the scarcity of robust empirical evidence on the impact of outsourcing *per se*. A few descriptive studies from the 1990s, which compared the crude NHS Audit scores across hospitals, suggested potentially worse performance among hospitals outsourcing cleaning services (Davies, 2010). These studies argued that outsourcing to private contractors led to poorer coordination between nursing staff and independent cleaners, especially as previous lines of accountability had been broken. However, the ability to evaluate these claims was limited by _ENREF_9a lack of data on rates of hospital-acquired infection. This has now changed, with the NHS's mandatory surveillance of MRSA, implemented in 2005 (Johnson et al., 2012), creating a set of comparative data over time. Under the new system, the MRSA rate is calculated as the number of MRSA bacteraemia reports from that Hospital Trust per 100,000 bed days (in the UK a Hospital Trust is a public entity that hospital operates facilities on one or more sites). Starting from October 2005, all Trusts in England were asked to submit data electronically, and in 2006 this system was further enhanced to provide data on possible sources of the MRSA bacteraemia, although this was only on voluntary basis. Until 2009 reports on MRSA bacteraemia rates in each acute Trust were published at six or 12 months interval; afterwards the reports were published on a monthly, quarterly and annual basis.

Here, for the first time to our knowledge, we test the hypothesis that outsourcing cleaning facilities is associated with greater incidence of MRSA, by linking newly available comparative data on its incidence with data on the provision of cleaning across English Acute Hospital Trusts.

## 2 Methods

### 2.1 Data sources

We linked data on MRSA incidence with patient reports of perceived hospital cleanliness, and health workers' reports of availability of handwashing facilities for 126 Acute Trusts. Data on hospital-borne MRSA incidence per 100,000 hospital bed-days were taken from Public Health England's annual reports (Public Health England, 2015). Data on patient-reported cleanliness were obtained from the Picker Institute NHS Patient Survey Programme (Care Quality Commission, 2010–2014) while data on handwashing facilities were from the Picker NHS National Staff Survey (Picker Institute Europe, 2010–2014). The two surveys are commissioned by NHS England from Picker Institute Europe. In the first, each Trust sends a questionnaire to 850 patients who have spent at least one night in the hospital between June and August each year. All the sampled patients are asked “In your opinion, how clean was the hospital room or ward (toilets and bathrooms) that you were (used) in? Very clean (excellent), fairly clean, not very clean, not clean at all”. In the NHS staff survey, each Trust selects a random group of staff (sample sizes will depend on the number of staff employed by the organisation from 600 to 850) to be interviewed. The survey asks all selected employees about their job, management, health/safety, and well-being in the Trust as well as their personal development. Here we are interested in a particular question “Are handwashing materials always available? Yes/No”. All data were for the years 2010–2014. Data on whether hospitals outsourced cleaning were obtained from Patient Environment Action Teams (2010-2)(Health & Social Care Information Centre, 2010–2014b) and Patient-Led Assessments of the Care Environment (2013-4) (Health & Social Information Centre, 2013–2014) (the name changed but collection practices did not). In practice, virtually all Trusts either fully outsourced or operated in-house cleaning services. Additional data on economic costs of cleaning per bed, staff numbers, patient mix and demographics, as well as size and services provided by the hospitals were taken from Estates Return Information Collection (ERIC) for the period 2010–2014 (Health & Social Care Information Centre, 2010-2014a). Table 1 in the web appendix provides further descriptive statistics for all variables used in the study.

Our initial sampling frame included all acute general hospital Trusts in England. We excluded single speciality orthopaedic, cardiac/ophthalmology/otolaryngology, gynaecology and paediatric hospitals given their atypical case mix (namely, Harefield, Royal National Orthopaedic, Royal National Throat, Nose and East Hospital, Papworth, Alder Hey, Robert Jones and Agnes Hunt Orthopaedic, Great Ormond, Moorefield Eye Hospital, Birmingham Children's Hospital, Heart of England NHS Foundation, Birmingham women's NHS foundation Trust and Sandwell and West Birmingham Hospital NHS Trust, and Royal Free Hampstead NHS Trust). Between 2010 and 2014 there were a total of 320 Acute Care Trusts, of which complete data existed for 201. It was not possible to track data over time in 119 Trusts because they changed identification codes during mergers. Of the 201, 140 report MRSA rates for the entire period. To avoid potential confounding from mixed service providers and switching (and numbers were too small to permit difference-in-difference analysis), we exclude a further four Trusts that use a combination of in-house and outsourced services and another four that changed from in-house to outsourcing (2) or vice-versa (2). Another four Trusts were removed because of small numbers or because they reported very high numbers (e.g. 7-fold higher than the median that indicated major outbreaks likely to have specific causes). Thus, our final analytical sample includes 126 acute Trusts. Of these 51 outsourced cleaning and 75 retained it in-house. Web appendix Fig. 1 further documents the sample inclusion criteria.

It is important to ascertain whether there were any pre-existing differences between hospitals that outsourced cleaning and those retaining it in-house, which might bias results, for example if hospitals with a worse cleaning record selectively outsourced it. Unfortunately, there are few sources of data that would allow such a comparison. One that does provide some insight is the dataset on hospital cleanliness, as assessed by the Healthcare Commission, from between three and five years prior to the data used in the main analysis, which start in 2010. We use these data to explore whether our results are consistent after adjusting for pre-existing differences in hospital sites, as measured by this indicator many years before the differences in out-sourcing (see web appendix Fig. 2 for more details).

### 2.2 Statistical modelling

We used multi-variate regression models to assess the association of outsourcing with MRSA incidence rates, as follows:

(1) $$MRSA_{it}=\alpha +\beta Outsource_{i}\;+\;\gamma Trust_{it}+\mu_{i}+n_{t}+\varepsilon_{it}\;$$

Here *i* is Trust and *t* is year. *MRSA* is the MRSA incidence rate per 100,000 hospital beds; *Outsource* is a dummy for whether the Trust outsourced cleaning services or retained them in-house; *Trust* is a series of variables controlling for Trust differences, including the number of beds in the Trusts and the average length of stay in the Trust; *μ* adjusts for four regional dummies (North, South, East, and West), and *n* is a set of year dummies to control for geo-spatial correlation, such as periods of MRSA outbreaks. *ε* is the error term.

To further adjust for potential confounding and facilitate comparability across Trusts, in a subsequent step we matched hospitals within geographic regions on dimensions of size (measured by number of hospital beds), complexity (measured as numbers of specialist and multiservice sites hospital within each Trust *i*) and case mix using propensity score matching (Rosenbaum and Rubin, 1983). Importantly, we match the two dimensions separately with respect to complexity, to take account of the possibility that differences in the number of specialist and multiservice sites might confound the results. Our ability to adjust for patient case mix is constrained by the absence of any severity measure based on diagnostic codes or something similar that predicts hospital acquired infection (as opposed to, for example and with caveats, the well-established case mix predictors of mortality). Propensity Score matching reduces potential confounding by comparing hospitals operating in similar regions, with matching size and complexity, but differing their management's choice of cleaning operation. It is used in policy evaluation because it reduces confounding compared with simple OLS models (Imbens, 2004). At this stage the 126 Trusts that had data on both MRSA rates in at least one year and sufficient information on complexity to enable matching were analysed. As a further robustness check we also implement coarsened exact matching (Iacus et al., 2011), which further address potential sources of residual confounding. The comparative advantage of coarsened exact matching vis-a-vis propensity score matching is that it ensures multivariate balancing between treated and control group.

All data and models were estimated using Stata version 13. All *t*-tests were two-tailed assuming unequal variances. Standard errors were bootstrapped and clustered by Trust to account for non-independence of sampling (Abadie and Imbens, 2009).

## 3 Results

### 3.1 Unadjusted comparison of outsource and in-house cleaning provision

**Fig. 1:**
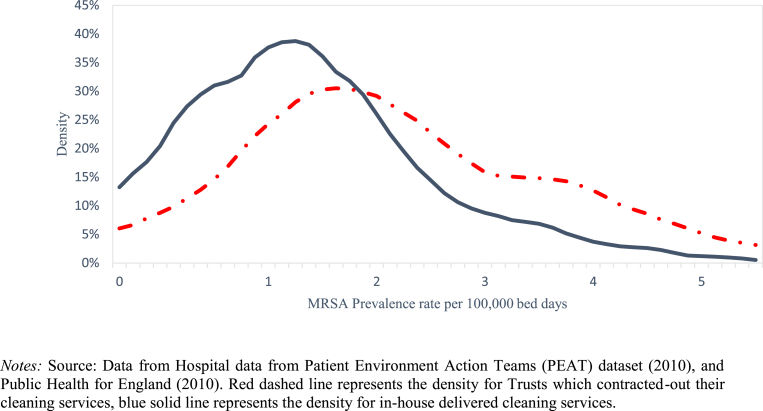
MRSA Incidence Rate by type of cleaning service in 2010.

Fig. 1 compares the pattern of MRSA incidence per 100,000 hospital bed-days in outsourced and in-house hospitals in 2010. The mean MRSA incidence in outsourced hospitals is 2.28 per 100,000 bed-days, almost 50% greater than the observed mean of 1.46 per 100,000 bed days in those that retained in-house cleaning (Stone et al.). Indeed, as shown in Fig. 3 in the web appendix, the entire MRSA risk distribution is greater in outsourced hospitals, which reflect the high levels of MRSA risk.

Next, we evaluated patient perceptions of cleanliness of bedrooms and bathrooms (web appendix Fig. 4a and b). Fewer patients in Trusts with outsourced services (57.6%) compared to in-house services (59.7%) described the cleanliness of the bedrooms as ‘excellent’ (*t*-test: 2.55, p = 0.01). We also observe a similar pattern for bathroom cleanliness (67.0% for outsourced hospitals compared with 68.5% for in-house hospitals; *t*-test = 2.04, p = 0.04).

In web appendix Fig. 5 we present the distribution of the percentage of staff who report access to hand-washing material across Trusts. 63.0% of staff who work in Trusts with outsourced cleaning services report that hand-washing materials are always available compared with 68.0% in Trusts with in-house cleaning (*t*-test: 3.47 p=<0.001).

### 3.2 Adjusted association of outsourcing with MRSA incidence rates

Table 1 shows the results of our statistical models, which can be interpreted as the average variation in MRSA incidence rate between Trusts which outsourced their cleaning services and those which retained their cleaning services in house. (In web appendix table 4, we also present the results using log-outcomes). Using simple OLS models we estimate that Trusts which outsourced their cleaning services tend to report on average 0.42 more cases of MRSA bacteraemia per 100,000 bed-days (95% CI: 0.24 to 0.61, *p*-value = 0.001). To translate this number into the original framework, we estimate the level of MRSA infection in two scenarios when cleaning services for the Trust *i* are outsourced vis-à-vis when they are provided in house. Accordingly, while outsourced Trusts will report an average rate of MRSA bacteraemia of to 1.44 cases per 100,000 bed days, their counterpart with in-house cleaning will report an average MRSA bacteraemia rate of 1.02.

Next, to adjust for differences due to potential observable confounding across hospitals, we estimated the association of outsourcing with MRSA, adjusting for hospital size, patient mix, and complexity. As shown Table 1, after correcting for these potentially confounding factors, we find that outsourcing is still associated with 0.22 more cases of MRSA bacteraemia per 100,000 bed-days (95% CI: 0.04 to 0.39, *p*-value = 0.01). Again, to translate our estimation into a measure that will be meaningful in the original framework, we estimate the level of MRSA infection in our two scenarios, setting all the other covariates at their median value. According to this model, while Trusts outsourcing cleaning will report a MRSA rate of 1.32 per 100,000 bed-days, their matched in house comparator will report an average rate of 1.10.

As an additional step, we matched hospitals within geographic regions of the UK and to the nearest-neighbour on size and complexity. It was not possible to match 34 of the 126 Trusts using this method (including 18 Trusts with in-house cleaning and 16 that outsourced it) because they were too different in size (in 18 cases) or complexity (in 12 cases) or in terms of propensity itself (based on the maximum permitted difference - i.e. the caliper - between observations) (4 cases), leaving a total of 92 matched Trusts (see web appendix Table 3, Table 3b for more details).

Table 1 further presents the results of the matched models. As anticipated, this yields a more precise estimate, with outsourcing now associated with 0.29 more cases of MRSA bacteraemia per 100,000 bed-days (95% CI: 0.17 to 0.37, *p*-value = 0.01).

Trusts outsourcing cleaning report an average rate of MRSA bacteraemia of 1.34 per 100,000 bed-days while their in-house counterparts report an average rate of 1.05 per 100,000 bed-days.

Finally, we implemented a Heckman selection model to assess the possibility of selection bias into outsourcing. We do not find clear evidence suggesting selection (IMR = 0.27, p = 0.38) (Table 1 column 4). The coefficient is not, however, statistically significant, mainly because standard errors tend to be large when the common support condition is not reached (Caliendo and Kopeinig, 2008).

Table 2-presents the estimation of the association between outsourcing of cleaning services on outcomes other than MRSA infection rates, adjusting the differences between in-house and outsourced cleaning procedure through propensity score matching, namely percentage of staff reporting ready access to hand-washing material (column 1), percentage of patients reporting excellent cleanliness for the bathroom they used (column 2). We present the results in terms of the average variation in MRSA incidence between Trusts which outsource their cleaning services and those which retain their cleaning services in house. The variation in percentage points is presented in web appendix table 5.

Our evidence indicates that in outsourced Trusts fewer people report ready access to hand-washing material (i.e. our proxy for the shortage of handwashing materials) by about 1.22% (95% CI --1.79% to −0.58%), and about 1 percentage points fewer patients reporting excellent cleanliness for the bathrooms (−0.45% percentage of patients reporting excellent cleanliness 95% CI: −0.46% to −0.44%0) and for rooms/wards (−0.76%, 95% CI: −0.01% to −0.002%). Translating the coefficients into the original framework, we find that while 61.3% of the outsourced Trusts will report having hand-washing material always available, their in-house peers will have 62.7%. The percentage of patients reporting excellent cleanliness in the bathrooms (rooms) are 58% (66.8%) and 58.49% (67.5%) respectively.

### 3.3 Comparing economic costs

Since one of the main arguments for outsourcing cleaning service in hospitals was to reduce costs, we also estimate the association between outsourcing of cleaning services on the cleaning cost per bed (see column 1 in Table 3) and cleaning personnel (column 2). The variation in percentage points is presented in web appendix table 6.

Our models estimate that outsourced Trusts have a lower cost of cleaning per bed of about £236 per bed per year (95% CI: £294 to -£172), and employ fewer cleaning staff, by about −0.006 people (95% CI: −0.008 to −0.001). Translating these coefficients into predictions, we find that the average cost per bed for Trusts that outsourced their cleaning services is about £2,894, while the average cost per bed for their in-house counterpart is about £3130. Here, adjusting for potential confounding factors appear to be particularly relevant, since the unadjusted comparison between the two average cost would have been misleading. With respect to the cleaning staff employed, we predict that outsourced Trusts would employ 0.126 staff per-bed, while in-house Trusts would employ 0.133 staff per-bed.

### 3.4 Robustness checks

We applied a series of sensitivity tests to our main statistical models, presented in web appendix table 7. The variation in percentage points is presented in web appendix table 8.

First, we restricted the sample to only those Trusts which had one hospital site (63% of the final sample – column 1). The results did not qualitatively differ (0.30 more cases of MRSA bacteraemia per 100,000 bed-days; 95% CI: 0.21 to 0.43). Second we used Coarsened Exact Matching (CEM) to re-estimate our matching models (Iacus et al., 2011), with similar results (0.30; 95% CI: 0.23 to 0.41). Third, to ensure that our results were not driven by the balanced panel, we ran a robustness test including all the Trusts observed at least once, and we find qualitatively similar results. Fourth, we check whether our results were driven by any pre-existing difference between outsourced and in-house Trusts. We replicated our analysis dropping two out of the five years, finding results consistent with our main ones. Fifth, to ensure that our results are not driven by the linear functional form we use a Poisson-model, again finding similar results (0.24, 95% CI: 0.19 0.65). Unfortunately, the models for counting data, such as Poisson models are limited to nonnegative numbers, therefore we cannot compute this robustness check for the log-outcomes. _ENREF_22.

## 4 Discussion

Outsourcing cleaning services was associated with significantly greater MRSA incidence, more reports that handwashing materials are not always available, and patient perceptions of less clean bathrooms and rooms/wards. However, economic costs per bed of outsourcing were also lower.

Our study has several limitations. First, we are currently using data only on Trusts whose MRSA incidence rate was recorded in all five years of the analysis. Attrition might be associated with a higher MRSA incidence rate, although we assume that this is not associated with the cleaning service type. We ran a robustness test including all the Trusts observed at least once, and we find qualitatively similar results. Outsourced Trusts tend to exhibit 0.35 (95 CI: 0.25 to 0.46) more cases of MRSA bacteraemia per 100,000 bed days. In the matching exercise, we were unable to include all Trusts because some lacked data on complexity and only 92 could be matched on these variables. Secondly, we only use data at Trust level, because of the lack of MRSA incidence data at site level. Since different sites within a single Trust might have adopted different cleaning-services, we might have misclassified the type of cleaning service. However, even when we restrict our models to include only single-site Trusts, we find similar results, suggesting that any bias created by misclassification of cleaning services is minor. Third, cleanliness is very likely to affect incidence rates of other hospital acquired infections but MRSA is currently the only infection for which we have comparable data. In addition, MRSA data are limited to infections that are detected in an individual's bloodstream and not all isolations. Hence our assessment of the problem is likely to be a substantial underestimate. Fourth, we would ideally wish to evaluate Trusts that switched cleaning services; however, in the period for which data were available, relatively few trusts switch, and a complicating factor is that these switches were likely to have occurred in relation to performance issues. However we can draw on the findings of a study that introduced an extra cleaner to two matched wards for six months each, using a crossover design, and found a 27% reduction in infections with MRSA, with the benefit disappearing after removal of the cleaner (S. J. Dancer et al., 2009). This is directly relevant to our finding that outsourced cleaning employs fewer staff. Fifth, we do not have any information on the screening practises used by the Trusts but there is no reason to believe that this would be systematically different between the in-house and the outsourced ones. Sixth, we did not have any data on staff-turnover or recruitment and/or sickness leave, which might be a good measure of both job-dissatisfaction and cleaning quality. Seventh, using data from several years before our study, we found no evidence that those Trusts outsourcing cleaning were systematically less clean, a possible cause of confounding by indication. However, caution is required as we cannot be sure that the Healthcare Commission data exclude a selection effect. Unfortunately, there are no other data that would be able to do so.

These findings have important implications. Although, from a narrow accounting perspective, Trusts outsourcing cleaning seem to incur lower costs of cleaning per bed, this is also associated with fewer staff and reduced reported availability of hand-washing material as well as an overall increased incidence of MRSA. However, it is not possible to conduct a full economic analysis because of an absence of comprehensive data on the nature and severity of the entire range of infections associated with poor cleaning, any additional deaths, the additional cost of treatment, and any associated costs, such as litigation. This is clearly an area for future research.

Notwithstanding these limitations, the fact that the antibiotic armamentarium is rapidly depleting means that our findings should be considered a reason for considerable concern.

## Figures and Tables

**Table 1:** Mean variation due to contracting-out cleaning services vis-a-vis retaining them in house on MRSA incidence rate.

	Incidence rate of MRSA infection			
	Bivariate association	Adjusted models	Propensity score matching	Heckman selection model
Mean variation due to contracting-out cleaning services vis-a-vis retaining them in house	0.42*** (0.09)	0.22** (0.09)	0.29*** (0.05)	0.26 (0.33)
*p*-value under the null hypothesis of no-selection bias	–	–	–	0.71
Number of Trust-years	582	582	446	582

Notes: Source: Data from Hospital data from Patient Environment Action Teams (PEAT) dataset (from 2010 till 2012), Patient-Led Assessments of the Care Environment (PLACE) (2013–2015), ERIC (Estates Return Information Collection) (2010–2015), NHS Inpatient Survey (2010–2014), NHS Staff Survey (2010–2014), and Public Health for England (2010–2014). Robust SE clustered at Trust level for models 1 and 2 and bootstrapped SE-values in parentheses (250 replications), stratifying by type of cleaning service, for models 3, 4 and 5. Coefficients represent average variation in MRSA incidence rate between Trust which outsource their cleaning services and those which retain their cleaning services in house.The dependent variable represents the incidence of MRSA infection at Trust level. Trust are matched through Matching (model 3) and their distribution are aligned by region, number of beds, number of specialist sites, number of multi sites. After having aligned the distribution we regress, through a linear model, the dependent variable on the number of beds, average length of stay, regional and year dummies.

*p < 0.05 **p < 0.01 ***p < 0.001.

**Table 2:** Association of contracting out cleaning services with other outcomes.

	Hand-washing availability Staff-Reported	Excellent cleanliness bathroom Patients reported	Excellent cleanliness room Patients reported
Mean variation due to contracting-out cleaning services vis-a’-vis retaining them in house	−1.22%*** (0.30)	−0.45%*** (0.003)	−0.76%*** (0.003)
Number of Trust-years	362	446	446

Notes: Source: Data from Hospital data from Patient Environment Action Teams (PEAT) dataset (from 2010 till 2012), Patient-Led Assessments of the Care Environment (PLACE) (2013–2015), ERIC (Estates Return Information Collection) (2010–2015), NHS Inpatient Survey (2010–2014), NHS Staff Survey (2010–2014), and Public Health for England (2010–2014). Bootstrapped SE-values in parentheses (250 replications), stratifying by type of cleaning service. Coefficients represent average variation in MRSA incidence rate between Trust which outsource their cleaning services and those which retain their cleaning services in house. The dependent variable represents: the percentage of staff reporting that hand-washing material is always available (column 1), percentage patients reporting excellent cleanliness of the bathroom they use (column 2) and percentage patients reporting excellent cleanliness of the room or ward they stayed (column 3). Trust are matched through Propensity Score Matching and their distribution are aligned by region, number of beds, number of specialist sites, number of multi sites. After having aligned the distribution we regress, through a linear model, the dependent variable on the number of beds, average length of stay, regional and year dummies.

*p < 0.05 **p < 0.01 ***p < 0.001.

**Table 3:** Association of contracting out cleaning services on economic cost outcomes.

	Cost per bed	Staff per bed
Mean variation due to contracting-out cleaning services vis-a-vis retaining them in house	-£236*** (33.7)	−0.01 p.*** (0.002)
Number of Trust-years	446	442

Notes: Source: Data from Hospital data from Patient Environment Action Teams (PEAT) dataset (from 2010 till 2012), Patient-Led Assessments of the Care Environment (PLACE) (2013–2015), ERIC (Estates Return Information Collection) (2010–2015), NHS Inpatient Survey (2010–2014), NHS Staff Survey (2010–2014), and Public Health for England (2010–2014). Bootstrapped SE-values in parentheses (250 replications), stratifying by type of cleaning service. Coefficients represent average variation in MRSA incidence rate between Trust which outsource their cleaning services and those which retain their cleaning services in house. The dependent variable represents: cost for cleaning (per-bed column 1, measured in £), staff employed for cleaning per-bed (column 2, measured in people per bed [p]).Trust are matched through Propensity Score Matching and their distribution are aligned by region, number of beds, number of specialist sites, number of multi sites. After having aligned the distribution we regress, through a linear model, the dependent variable on the number of beds, average length of stay, regional and year dummies.

*p < 0.05 **p < 0.01 ***p < 0.001.

## References

[bib1] Abadie A., Imbens G.W. (2009). Matching on the Estimated Propensity Score.

[bib2] BBC News (2008). End Private Cleaning in NHS Call. http://news.bbc.co.uk/1/hi/health/7372992.stm.

[bib3] Caliendo M., Kopeinig S. (2008). Some practical guidance for the implementation of propensity score matching. J. Econ. Surv..

[bib4] Care Quality Commission P.I.E (2010-2014). NHS Patient Survey Programme (Acute Trusts: Adult Inpatients Survey). In C.Q.C. National Health Service.

[bib5] Chan P., Dipper A., Kelsey P., Harrison J. (2010). Newspaper reporting of meticillin-resistant Staphylococcus aureus and ‘the dirty hospital’. J. Hosp. Infect..

[bib6] Conservative Party (1983). General Election Manifesto. http://www.margaretthatcher.org/document/110859.

[bib7] Daily Record (2011). Number of Cases of Killer Hospital Bug clostridium difficile in Scotland's Hospitals Have Dropped 37% in a Year. http://www.dailyrecord.co.uk/news/health/number-of-cases-of-killer-hospital-bug-1099571.

[bib8] Dancer S.J. (2008). Importance of the environment in meticillin-resistant Staphylococcus aureus acquisition: the case for hospital cleaning. Lancet Infect. Dis..

[bib9] Dancer S. (2009). The role of environmental cleaning in the control of hospital-acquired infection. J. Hosp. Infect..

[bib10] Dancer S.J., White L.F., Lamb J., Girvan E.K., Robertson C. (2009). Measuring the effect of enhanced cleaning in a UK hospital: a prospective cross-over study. BMC Med..

[bib11] Davies S. (2009). Making the Connections: Contract Cleaning and Infection Control.

[bib12] Davies S. (2010). Fragmented management, hospital contract cleaning and infection control. Policy & Polit..

[bib13] European Federation of Public Service Unions (2011). UK: Hospital Cleaning Brought in House in Scotland. http://www.epsu.org/a/7405.

[bib14] Greaves F., Pape U.J., King D., Darzi A., Majeed A., Wachter R.M. (2012). Associations between Web-based patient ratings and objective measures of hospital quality. Archives Intern. Med..

[bib15] Health & Social Care Information Centre, Health D.o. (2010). Estates Return Information Collection.

[bib16] Health & Social Care Information Centre, Health D.o. (2010). Patient Environment Assessment Team (PEAT).

[bib17] Health & Social Information Centre, Health D.o. (2013-2014). Patient-led Assessments of the Care Environment (PLACE), England.

[bib18] Iacus S.M., King G., Porro G. (2011). Causal inference without balance checking: coarsened exact matching. Polit. Anal..

[bib19] Imbens G.W. (2004). Nonparametric estimation of average treatment effects under exogeneity: a review. Rev. Econ. statistics.

[bib20] Johnson A.P. (2011). Methicillin-resistant Staphylococcus aureus: the european landscape. J. Antimicrob. Chemother..

[bib21] Johnson A.P., Davies J., Guy R., Abernethy J., Sheridan E., Pearson A. (2012). Mandatory surveillance of methicillin-resistant Staphylococcus aureus (MRSA) bacteraemia in England: the first 10 years. J. Antimicrob. Chemother..

[bib22] Nathwani D., Sneddon J., Patton A., Malcolm W. (2012). Antimicrobial stewardship in Scotland: impact of a national programme. Antimicrob. Resist. Infect. control.

[bib23] Picker Institute Europe (2010-2014). National Health Service National Staff Survey.

[bib24] Public Health England (2015). Financial Year Counts and Rates of Meticillin Resistant Staphylococcus aureus (MRSA) Bacteraemia from – Trust Apportioned Cases Only.

[bib25] Rosenbaum P.R., Rubin D.B. (1983). The central role of the propensity score in observational studies for causal effects. Biometrika.

[bib26] Sroka S., Gastmeier P., Meyer E. (2010). Impact of alcohol hand-rub use on meticillin-resistant Staphylococcus aureus: an analysis of the literature. J. Hosp. Infect..

[bib27] Stone S.P., Fuller C., Savage J., Cookson B., Hayward A., Cooper B. (2012). Evaluation of the national Cleanyourhands campaign to reduce Staphylococcus aureus bacteraemia and Clostridium difficile infection in hospitals in England and Wales by improved hand hygiene: four year, prospective, ecological, interrupted time series study. BMJ.

[bib28] Trucano M., Kaldenberg D. (2007). The Relationship between Patient Perceptions of Hospital Practices and Facility Infection Rates: Evidence from Pennsylvania Hospitals.

[bib29] Washer P., Joffe H. (2006). The “hospital superbug”: social representations of MRSA. Soc. Sci. Med..

