# Supplementary material for: Outsourcing cleaning services increases MRSA incidence: Evidence from 126 english acute trusts
Source: Soc Sci Med. 2017 Feb;174:64–9. doi: 10.1016/j.socscimed.2016.12.015 (PMC5267843; doi:10.1016/j.socscimed.2016.12.015)
Supplement: Supplementary Figs and Tables [file mmc1.docx]

**Web Appendix**

**Figures and Tables**

Figure 1: Sample Selection

Figure 2: Comparison of quality of services performance of the type of cleaning service between 2005/6 and 2007/8

Figure 3: Cumulative Distribution Function by type of cleaning service in 2010

Figure 4: Percentage of Patients Reporting Excellent Cleanliness, Rooms and Bathrooms

Figure 5: Percentage of Staff Reporting to Have Access to Handwashing Material by type of cleaning service in 2010

Figure 6: MRSA Incidence Rate by type of cleaning service in 2010

Table 1: Descriptive statistics by type of cleaning service

Table 2: t-test comparison between in-house and contracted-out Trust hospitals

Table 3: Baseline characteristics of trusts which outsourced their cleaning service, compared with those which do not, before and after matching (propensity score) comparison

Table 3b: Estimation of the propensity score matching, logit

Table 4: Robustness checks: Association of contracting out cleaning services with MRSA incidence rates, using log-outcomes, values represents percentages

Table 5: Association of contracting out cleaning services with other outcomes, marginal effects

Table 6: Association of contracting out cleaning services on economic cost outcomes, marginal effects

Table 7: Robustness checks: Association of contracting out cleaning services with MRSA incidence rates

Table 8: Robustness checks: Association of contracting out cleaning services with MRSA incidence rates, marginal effects

Figure 1: Sample Selection


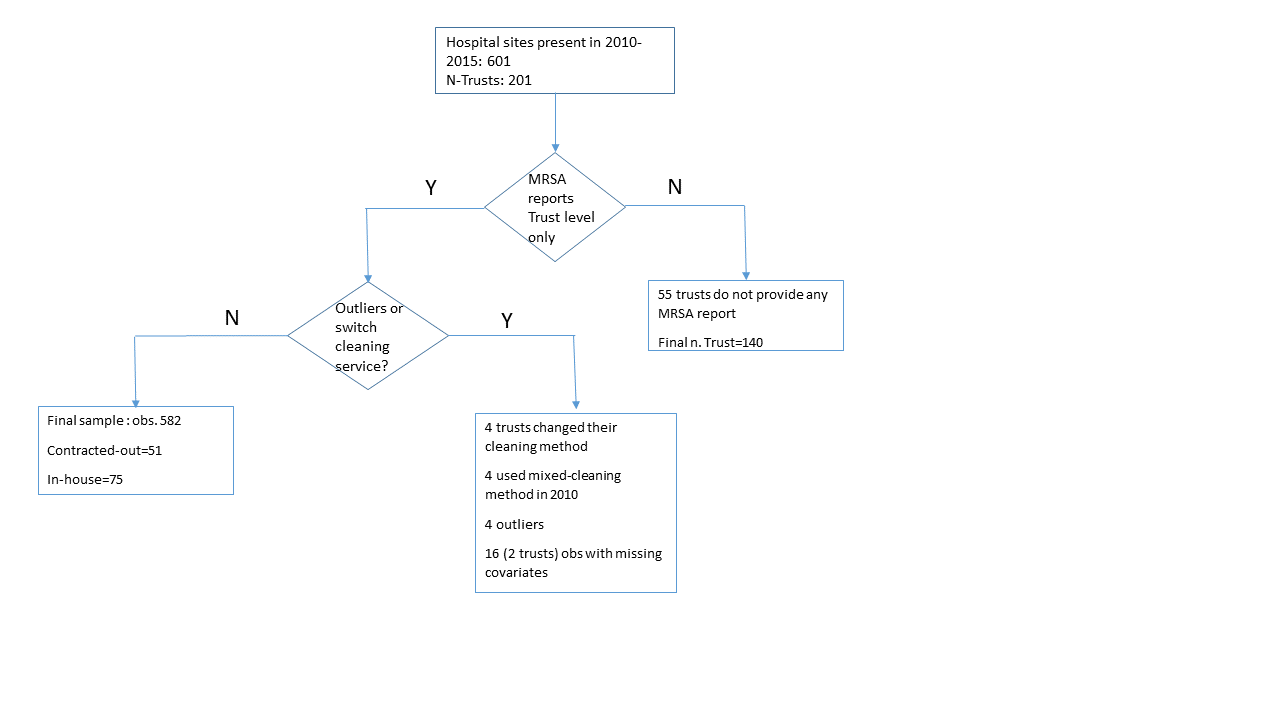


Figure 2: Comparison of quality of services performance of the type of cleaning service between 2005/6 and 2007/8

*Notes:* Source: Data on type of cleaning come from Patient Environment Action Teams (PEAT) dataset (2010), Evaluation data come from “The annual check 2007/8”.

Figure 3: Cumulative Distribution Function by type of cleaning service in 2010

*Notes:* Source: Data from Hospital data from Patient Environment Action Teams (PEAT) dataset (2010), and Public Health for England (2010). Red dash line represents the density for Trusts which contracted-out their cleaning services, blue solid line represents the density for in-house delivered cleaning services.

Figure 4: Percentage of Patients Reporting Excellent Cleanliness

1. Rooms


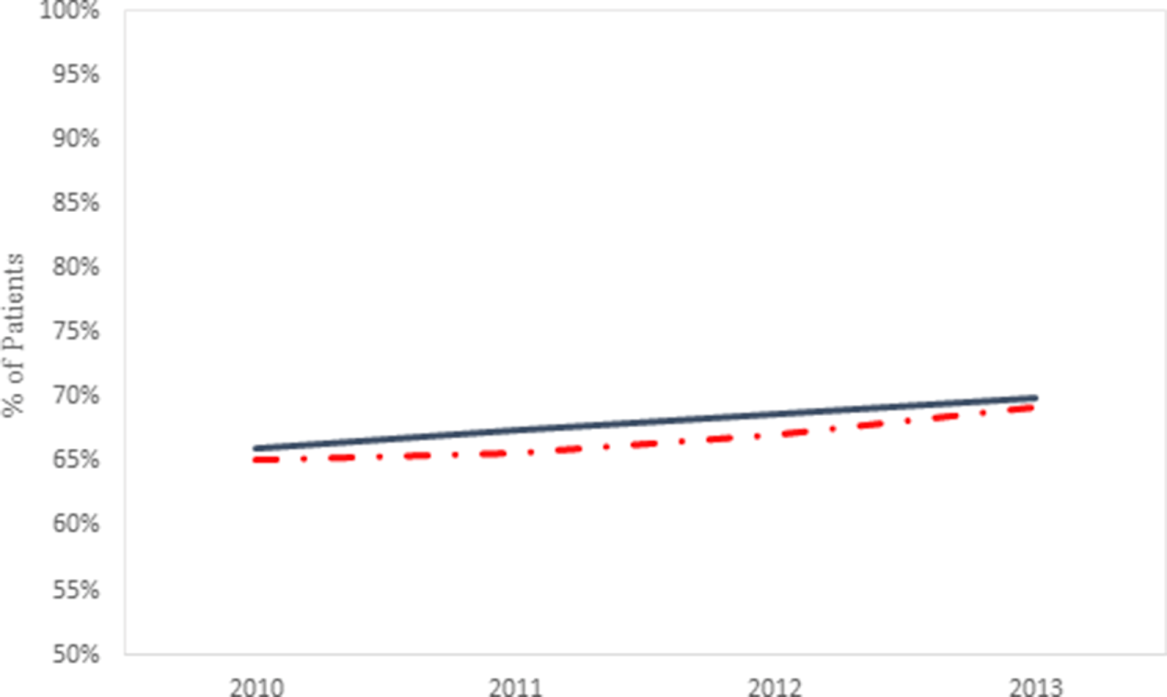


1. Bathrooms

*Notes:* Source: Data from Hospital data from Patient Environment Action Teams (PEAT) dataset (from 2010 till 2012), Patient-Led Assessments of the Care Environment (PLACE) (2013-2015), NHS Inpatient Survey (2010-2014). Red dashed line represents trusts which contracted-out their cleaning services, blue solid line represents trusts which deliver in-house their cleaning services.

Figure 5: Percentage of Staff Reporting to Have Access to Handwashing Material by type of cleaning service in 2010, data for 126 Acute Trusts

*Notes:* Source: Data from NHS Staff Survey (2010). Red dashed line represents trusts which contracted-out their cleaning services, blue solid line represents trusts which deliver in-house their cleaning services.

Figure 6: MRSA trend by type of cleaning service

*Notes:* Source: Data from Hospital data from Patient Environment Action Teams (PEAT) dataset (from 2010 till 2012), Patient-Led Assessments of the Care Environment (PLACE) (2013-2015), ERIC (Estates Return Information Collection) (2010-2015), NHS Inpatient Survey (2010-2014), NHS Staff Survey (2010-2014), and Public Health for England (2010-2014). Red dashed line represents the trend for trusts which contracted-out their cleaning services, blue solid line represents the trend for in-house delivered cleaning services.

Table 1: Descriptive statistics

| Variable | Mean | Min. | Max. | Source |
| --- | --- | --- | --- | --- |
| % of Patients reporting Excellent in Bathroom Cleanliness (Patient) | 58.9  (9.11) | 31.7 | 88.9 | NHS Inpatient Survey (2010-2015) |
| % % of Patients reporting Excellent in Room Cleanliness (Patient) | 67.9  (8.07) | 45.5 | 93.8 | NHS Inpatient Survey (2010-2015) |
| Mean Length of Stay (days) | 6.02  (1.05) | 2.45 | 9.77 | NHS Inpatient Survey (2010-2014) |
| Number of Beds | 1333  (677 ) | 120 | 3986 | Estates Return Information Collection (ERIC) (2010-2015) |
| Cost of cleaning (£ per bed) | 3087  (1031) | 412 | 9549 | Own elaboration from Estates Return Information Collection (ERIC) (2010-2015) |
| Staff whole time equivalent (WTE) cleaning (n. people per bed) | 0.13  (0.05) | 0 | 0.52 | Own elaboration from Estates Return Information Collection (ERIC) (2010-2015) |
| % of staff had a training infection control in the last 12 months | 77.2  (8.62) | 53 | 96.6 | NHS Staff Survey (2010 -2014) |
| % of Staff reporting to have hand wash material | 62.8  (8.45) | 37.7 | 81.9 | NHS Staff Survey (2010 -2014) |
| N. of Specialist Sites (Total number of sites that are being reported on that predominantly undertake a specialist function, inclusive of Radiotherapy, Dental Hospital, Maternity Hospital, Children’s Hospital, Oncology, Cardiology and Secure Unit for mental health) | 0.23  (0.62) | 0 | 4 | Estates Return Information Collection (ERIC) (2010-2015) |
| N. of Multiservice Sites (Total number of sites that are being reported on that are hospitals providing multi-service functions, including single speciality, acute services, mental health and community services. Such sites must provide at least two differing service functions each with gross internal floor area representing more than 20% of the total gross internal site floor area for the whole site.) | 0.18  (0.56) | 0 | 6 | Estates Return Information Collection (ERIC) (2010-2015) |
| MRSA incidence Rate | 1.20  (1.06) | 0 | 5.6 | Public Health for England (2010 -2014) |
| Total obs. (time-trust)582 | | | | |

*Notes:* Source: Data from Hospital data from Patient Environment Action Teams (PEAT) dataset (from 2010 till 2012), Patient-Led Assessments of the Care Environment (PLACE) (2013-2015), ERIC (Estates Return Information Collection) (2010-2015), NHS Inpatient Survey (2010-2014), NHS Staff Survey (2010-2014), and Public Health for England (2010-2014). Standard deviation in brackets.

Table 2: *t*-test comparison between in-house and contracted-out Trust hospitals

| Variable | In-House  (N-Trusts=75) | Contracted-out  (N-Trusts=51) | *t*-test Comparison between in-House and Contracted-out | P-value |
| --- | --- | --- | --- | --- |
| % of Patients reporting bathrooms have excellent cleanliness | 59.7  (7.87) | 57.6  (10.7) | 2.55 | 0.01 |
| % of Patients reporting rooms have excellent cleanliness (Patient) | 68.5  (6.98) | 67.0  (9.50) | 2.04 | 0.04 |
| Mean Length of Stay (days) | 5.97  (0.95) | 6.10  (1.18) | -1.43 | 0.15 |
| Number of Beds | 1304  (648) | 1377  (720) | -1.24 | 0.22 |
| Cost of cleaning (£ per bed) | 3046  (961) | 3151  (1131) | -1.16 | 0.25 |
| Staff whole time equivalent (WTE) cleaning (n. people per bed) | 0.13  (0.04) | 0.14  (0.06) | -1.95 | 0.05 |
| % of Staff had a training infection control in the last 12 months | 77.8  (8.27) | 76.4  (9.10) | 1.88 | 0.06 |
| % of Staff reporting to have hand wash material | 64.7  (7.71) | 60.0  (8.77) | 5.92 | <0.001 |
| Number of Specialist sites | 0.16  (0.53) | 0.34  (0.74) | -3.17 | <0.001 |
| Number Multiservice sites | 0.21  (0.64) | 0.13  (0.40) | 1.81 | 0.07 |
| MRSA incidence rate per 100,000 bed-day | 1.03  (0.88) | 1.46  (1.24) | -4.61 | <0.001 |

*Notes:* Source: Data from Hospital data from Patient Environment Action Teams (PEAT) dataset (from 2010 till 2012), Patient-Led Assessments of the Care Environment (PLACE) (2013-2015), ERIC (Estates Return Information Collection) (2010-2015), NHS Inpatient Survey (2010-2014), NHS Staff Survey (2010-2014) and Public Health for England (2010-2014).

Table 3: Baseline characteristics of trusts which outsourced their cleaning service, compared with those which do not, before and after matching (propensity score) comparison. Values are numbers (percentages) unless stated otherwise.

| Characteristic | Before Propensity Score Matching | | | After Propensity Score Matching | | |
| --- | --- | --- | --- | --- | --- | --- |
|  | In-House Cleaning Service (n=362 obs.) | Outsourced Cleaning service (n=228 obs.) | T-statistic difference | In-House Cleaning Service (n=279 obs.) | Outsourced Cleaning service (n=165 obs.) | T-statistic difference |
|  | Mean (S.D.) | Mean  (S.D.) | T-statistic | Mean (S.D.) | Mean  (S.D.) | T-statistic |
| Location | | | | | | |
| Midlands | 0.32 | 0.18 | 3.91 | 0.37 | 0.21 | 3.87 |
|  | (0.47) | (0.39) |  | (0.49) | (0.41) |  |
| South East of England | 0.26 | 0.28 | -0.63 | 0.30 | 0.32 | -0.50 |
|  | (0.44) | (0.45) |  | (0.46) | (0.47) |  |
| London | 0.05 | 0.27 | -7.24 | 0.05 | 0.21 | -4.77 |
|  | (0.21) | (0.45) |  | (0.22) | (0.41) |  |
| North of England | 0.37 | 0.26 | 2.90 | 0.28 | 0.26 | 0.49 |
|  | (0.48) | (0.44) |  | (0.45) | (0.44) |  |
| Complexity |  |  |  |  |  |  |
| N. of Specialist Sites | 0.16 | 0.34 | -3.17 | 0.09 | 0.10 | -0.39 |
|  | (0.53) | (0.74) |  | (0.31) | (0.33) |  |
| Multiservice | 0.21 | 0.13 | 1.81 | 0.08 | 0.12 | -1.15 |
|  | (0.64) | (0.40) |  | (0.35) | (0.41) |  |
| Capacity |  |  |  |  |  |  |
| N. of Beds | 1304 | 1377 | -1.23 | 1243 | 1299 | -1.05 |
|  | (647) | (720) |  | (555) | (546) |  |

*Notes:* Source: Data from Hospital data from Patient Environment Action Teams (PEAT) dataset (from 2010 till 2012), Patient-Led Assessments of the Care Environment (PLACE) (2013-2015), ERIC (Estates Return Information Collection) (2010-2015), NHS Inpatient Survey (2010-2014), NHS Staff Survey (2010-2014), and Public Health for England (2010-2014).

Table 3b: Estimation of the propensity score matching, logit

|  | Probability of Outsource Cleaning Services |
| --- | --- |
| Size(n. of beds in the hospital) | 0.0003**  (0.0001) |
| N. of specialist sties (complexity) | 0.41**  (0.17) |
| N. of multi-service sites (complexity) | -0.50**  (0.21) |
|  |  |
| Regional Fixed-Effect | Yes |
| Number of Trust-years | 582 |

*Notes:* Source: Data from Hospital data from Patient Environment Action Teams (PEAT) dataset (from 2010 till 2012), Patient-Led Assessments of the Care Environment (PLACE) (2013-2015), ERIC (Estates Return Information Collection) (2010-2015), NHS Inpatient Survey (2010-2014), NHS Staff Survey (2010-2014), and Public Health for England (2010-2014).

Table 4: Robustness checks: Association of contracting out cleaning services with MRSA incidence rates, using log-outcomes, values represent marginal effects

|  | Incidence rate of MRSA infection | | | |
| --- | --- | --- | --- | --- |
|  | Bivariate Association | Adjusted Models | Propensity Score Matching | Heckman selection model |
| Association of contracting out cleaning services (%) | 22.8%**  (6.81) | 14.0%*  (6.27) | 15.3%***  (3.23) | 12.5%  (26.7) |
|  |  |  |  |  |
| p-value under the null hypothesis of no-selection bias | __ | __ | __ | 0.96 |
|  |  |  |  |  |
| Number of Trust-years | 582 | 582 | 446 | 582 |

*Notes:* Source: Data from Hospital data from Patient Environment Action Teams (PEAT) dataset (from 2010 till 2012), Patient-Led Assessments of the Care Environment (PLACE) (2013-2015), ERIC (Estates Return Information Collection) (2010-2015), NHS Inpatient Survey (2010-2014), NHS Staff Survey (2010-2014), and Public Health for England (2010-2014). Robust SE clustered at Trust level for models 1 and 2 and bootstrapped SE-values in parentheses (250 replications), stratifying by type of cleaning service, for models 3, 4 and 5. Coefficients represent semi-elasticities. The dependent variable represents the logarithm of MRSA incidence rate at Trust level. Trust are matched through Matching (model 3) and their distribution are aligned by region, number of beds, number of specialist sites, number of multi sites. After having aligned the distribution we regress, through a linear model, the dependent variable on the number of beds, average length of stay, regional and year dummies.

* *p* < 0.05 ** *p* < 0.01 *** *p* < 0.001

Table 5: Association of contracting out cleaning services with other outcomes, using log-outcomes, values represent marginal effects

|  | Hand-washing availability  Staff-Reported | Excellent Cleanliness Bathroom  Patients reported | Excellent Cleanliness Room  Patients reported |
| --- | --- | --- | --- |
| Association of contracting out cleaning services (%) | -2.27%***  (0.49) | -0.90%*  (0.45) | -1.28%**  (0.44) |
|  |  |  |  |
| Number of Trust-years | 362 | 446 | 446 |

*Notes:* Source: Data from Hospital data from Patient Environment Action Teams (PEAT) dataset (from 2010 till 2012), Patient-Led Assessments of the Care Environment (PLACE) (2013-2015), ERIC (Estates Return Information Collection) (2010-2015), NHS Inpatient Survey (2010-2014), NHS Staff Survey (2010-2014), and Public Health for England (2010-2014). Bootstrapped SE-values in parentheses (250 replications), stratifying by type of cleaning service. Coefficients represent semi-elasticities. The dependent variable represents the logarithm of: availability of hand-washing material (column 1), and percentage patients reporting excellent cleanliness of the bathroom (column 2). Trust are matched through Propensity Score Matching and their distribution are aligned by region, number of beds, number of specialist sites, number of multi sites. After having aligned the distribution we regress, through a linear model, the dependent variable on the number of beds, average length of stay, regional and year dummies..

* *p* < 0.05 ** *p* < 0.01 *** *p* < 0.001

Table 6: Association of contracting out cleaning services on economic cost outcomes, using log-outcomes, values represent marginal effects

|  | Cost per Bed | Staff per Bed |
| --- | --- | --- |
| Change associated with contracting out cleaning services (%) | -6.93%***  (1.24) | -5.86%***  (1.38) |
|  |  |  |
| Number of Trust-years | 446 | 442 |

Notes: Source: Data from Hospital data from Patient Environment Action Teams (PEAT) dataset (from 2010 till 2012), Patient-Led Assessments of the Care Environment (PLACE) (2013-2015), ERIC (Estates Return Information Collection) (2010-2015), NHS Inpatient Survey (2010-2014), NHS Staff Survey (2010-2014), and Public Health for England (2010-2014). Bootstrapped SE-values in parentheses (250 replications), stratifying by type of cleaning service. Coefficients represent semi-elasticities. The dependent variable represents the logarithm of: cost for cleaning (per-bed column 1), staff employed for cleaning per-bed (column 2).Trust are matched through Propensity Score Matching and their distribution are aligned by region, number of beds, number of specialist sites, number of multi sites. After having aligned the distribution we regress, through a linear model, the dependent variable on the number of beds, average length of stay, regional and year dummies.

* p < 0.05 ** p < 0.01 *** p < 0.001

Table 7: Robustness checks: Association of contracting out cleaning services with MRSA incidence rates

| Incidence rate of MRSA infection | | | | | |
| --- | --- | --- | --- | --- | --- |
|  | Only trusts with 1 site, All Covariates | Coarsened Exact Matching, All Covariates | Using an unbalanced panel | Using sub-sample of our data  (4) | Using a Poisson Model |
|  | (1) | (2) | (3) |  | (5) |
| Mean variation due to contracting-out cleaning services vis-a’-vis retaining them in house | 0.30***  (0.06) | 0.30***  (0.05) | 0.35***  (0.05) | 0.38***  (0.07) | 0.24***  (0.04) |
|  |  |  |  |  |  |
| Number of Trust-years | 272 | 473 | 438 | 279 | 446 |

*Notes:* Source: Data from Hospital data from Patient Environment Action Teams (PEAT) dataset (from 2010 till 2012), Patient-Led Assessments of the Care Environment (PLACE) (2013-2015), ERIC (Estates Return Information Collection) (2010-2015), NHS Inpatient Survey (2010-2014), NHS Staff Survey (2010-2014), and Public Health for England (2010-2014). Bootstrapped SE-values in parentheses (250 replications), stratifying by type of cleaning service. Coefficients represent the average variation due to contracting-out cleaning services vis-a’-vis retaining them in house. The dependent variable represents the MRSA incidence rate at Trust level. Trust are matched through Matching and their distribution are aligned by region, number of beds, number of specialist sites, and number of multiservice sites. After having aligned the distribution we regress, through a linear model, the dependent variable on the number of beds, average length of stay, regional and year dummies.

* *p* < 0.05 ** *p* < 0.01 *** *p* < 0.001

Table 8: Robustness checks: Association of contracting out cleaning services with MRSA incidence rates, values represent percentages

| Incidence rate of MRSA infection | | | | |
| --- | --- | --- | --- | --- |
|  | Only trusts with 1 site, All Covariates | Coarsened Exact Matching, All Covariates | Using an unbalanced panel | Using sub-sample of our data  (4) |
|  | (1) | (2) | (3) |  |
| Association of contracting out cleaning services (%) | 9.55%**  (3.31) | 15.7%*  (6.89) | 14.6%***  (3.44) | 13.9%***  (4.39) |
|  |  |  |  |  |
| Number of Trust-years | 272 | 473 | 438 | 279 |

*Notes:* Source: Data from Hospital data from Patient Environment Action Teams (PEAT) dataset (from 2010 till 2012), Patient-Led Assessments of the Care Environment (PLACE) (2013-2015), ERIC (Estates Return Information Collection) (2010-2015), NHS Inpatient Survey (2010-2014), NHS Staff Survey (2010-2014), and Public Health for England (2010-2014). Bootstrapped SE-values in parentheses (250 replications), stratifying by type of cleaning service. Coefficients represent semi-elasticities. The dependent variable represents the logarithm of the MRSA incidence rate at Trust level. Trust are matched through Matching and their distribution are aligned by region, number of beds, number of specialist sites, and number of multiservice sites. After having aligned the distribution we regress, through a linear model, the dependent variable on the number of beds, average length of stay, regional and year dummies.

* *p* < 0.05 ** *p* < 0.01 *** *p* < 0.001
